# Nanoscale probing of image-dipole interactions in a metallic nanostructure

**DOI:** 10.1038/ncomms7558

**Published:** 2015-03-19

**Authors:** Chad Ropp, Zachary Cummins, Sanghee Nah, John T. Fourkas, Benjamin Shapiro, Edo Waks

**Affiliations:** 1 Department of Electrical and Computer Engineering and Institute for Research in Electronics and Applied Physics, University of Maryland, College Park, Maryland 20742, USA; 2 Fischell Department of Bioengineering and the Institute for Systems Research, University of Maryland, College Park, Maryland 20742, USA; 3 Department of Chemistry and Biochemistry, University of Maryland, College Park, Maryland 20742, USA; 4 Institute for Physical Science and Technology, University of Maryland, College Park, Maryland 20742, USA; 5 Joint Quantum Institute, National Institute of Standards and Technology, University of Maryland, College Park, Maryland 20742, USA

## Abstract

An emitter near a surface induces an image dipole that can modify the observed emission intensity and radiation pattern. These image-dipole effects are generally not taken into account in single-emitter tracking and super-resolved imaging applications. Here we show that the interference between an emitter and its image dipole induces a strong polarization anisotropy and a large spatial displacement of the observed emission pattern. We demonstrate these effects by tracking the emission of a single quantum dot along two orthogonal polarizations as it is deterministically positioned near a silver nanowire. The two orthogonally polarized diffraction spots can be displaced by up to 50 nm, which arises from a Young’s interference effect between the quantum dot and its induced image dipole. We show that the observed spatially varying interference fringe provides a useful measure for correcting image-dipole-induced distortions. These results provide a pathway towards probing and correcting image-dipole effects in near-field imaging applications.

## Abstract (web summary)

An emitter near a surface induces an image dipole that alters the emission pattern and creates errors in single-particle imaging applications. Here, Ropp *et al.* show that an image dipole can distort the polarization and measured position of an emitter, and that these distortions can be corrected.

## Graphical abstract

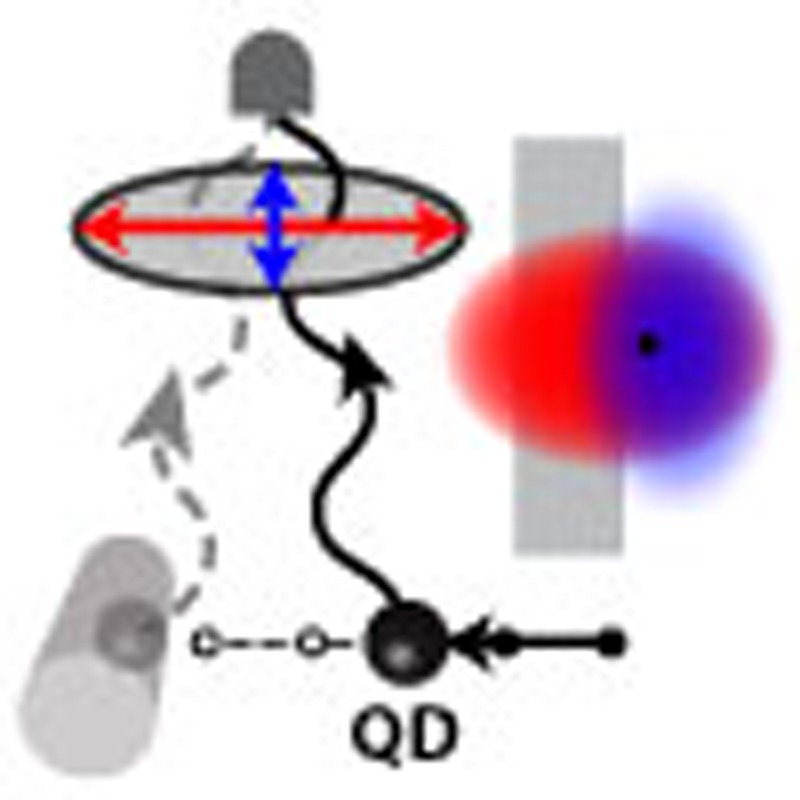

Single quantum emitters are ideal probes for studying the physics of nanoscopic systems. The small size of these emitters allows them to resolve nanoscale features with high resolution^1^,^2^,^3^. In recent years, optical tracking of single emitters has emerged as a powerful method for imaging and sensing nanoscale structures. In optical tracking, the centroid location and shape of the emitter’s diffraction spot determine its position to higher precision than the diffraction limit^4^,^5^, enabling imaging and probing with a spatial resolution of a few nanometres^6^. Applications of single-emitter tracking have included measuring local field enhancement of plasmonic nanostructures^7^,^8^,^9^,^10^, probing surface-enhanced Raman scattering^11^,^12^ and studying interfacial chemical reactions^13^,^14^.

The above applications rely on the ability to track an emitter near a surface. But, proximity to a surface can complicate single-emitter tracking^15^,^16^. Near the surface of a target object, the emitter’s local electric field can induce oscillating currents that act as a secondary radiation source, which is often referred to as an image dipole. At a planar metal or dielectric interface, the effect of an image dipole on the radiative intensity of an emitter is well understood^17^,^18^,^19^,^20^,^21^,^22^,^23^,^24^,^25^. For non-planar boundary conditions, however, image dipoles can lead to much more complex effects. These effects can be particularly difficult to quantify in nanoscale objects because of the presence of other interactions such as coupling to guided surface plasmon polaritons^21^, local excitation enhancement^26^, scattering from hot spots^27^,^28^,^29^ and antenna radiation^30^,^31^.

In this article, we show that the interference between an emitter and its image dipole in a nanostructure can induce substantial polarization-dependent changes to both the intensity and position of the emitter’s far-field diffraction spot. This phenomenon can have a major impact on the accuracy of single-particle tracking. We experimentally demonstrate these effects by deterministically positioning an individual quantum dot in the vicinity of a silver nanowire and tracking the emission simultaneously along two orthogonal polarizations. Interference between the quantum dot and the image dipole causes the emission to become preferentially polarized. Furthermore, the diffraction spots measured at polarizations parallel and perpendicular to the nanowire can be displaced by as much as 50 nm, which is much larger than the expected accuracy of single-emitter tracking techniques^6^. This position displacement occurs even at distances exceeding 200 nm from the wire surface, and is the result of a Young’s interference effect between the emitter and its image dipole. We use the interference fringe to correct for the effects of the image-dipole distortions on the measured nanowire local density of states (LDOS). The corrected LDOS shows significantly improved agreement with numerical full-wave calculations. These results provide a better fundamental understanding of image-dipole effects in nanostructures and offer a promising route to improve the accuracy of near-field probing and sensing applications.

## Results

### Image dipole-induced modification of dipole emission

**Figure 1:**
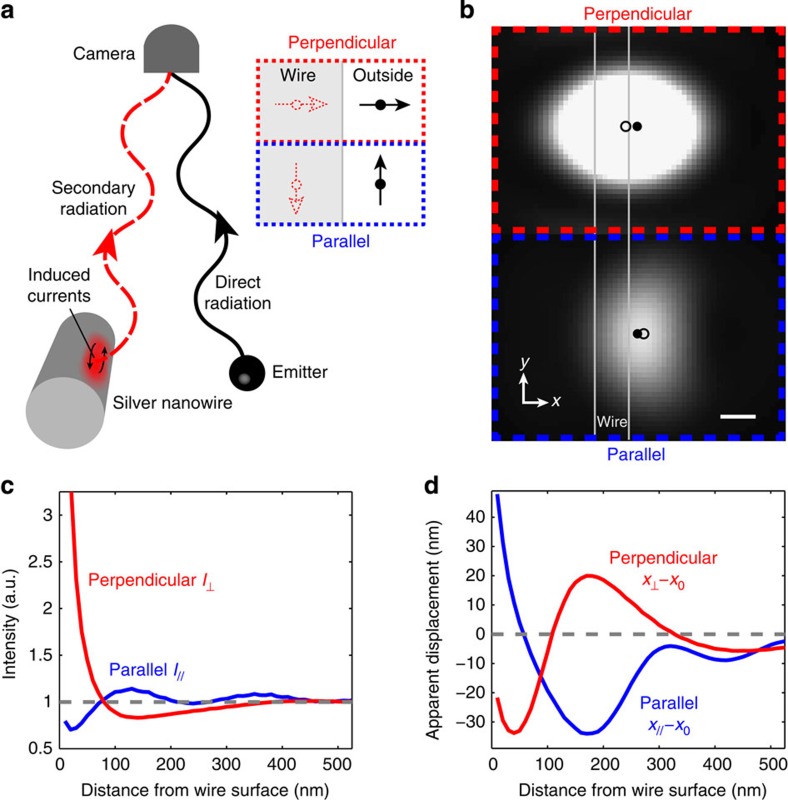
Image-dipole interference. (**a**) Illustration of interference between an emitter and secondary radiation from the nanowire. The emitter induces currents in the silver nanowire that radiate into the far field and interfere with the direct emitter radiation. Near the wire surface, modification to the far-field image can be described using an image-dipole model, as illustrated in the right inset, for dipoles oriented perpendicular (top) and parallel (bottom) to the surface. (**b**) Calculated far-field diffraction spots from an isotropic emitter located 30 nm from the wire surface (outlined in white) for emission polarized along the perpendicular (red) and parallel (blue) directions. Interference with the image dipole leads to differences in the intensities and displacement of the centroid positions (open circle) relative to the emitter position (closed circle). The coordinate system is shown at the bottom left. Scale bar, 100 nm. (**c**) Calculated intensity as a function of the distance of an isotropic emitter from the wire surface for the field polarized along the perpendicular (red) and parallel (blue) polarization directions. These curves have been normalized by the emitted intensity far from the wire. (**d**) The displacement of the diffraction spot position relative to the emitter position as a function of emitter distance from the wire surface for different emission polarizations.

Figure 1a illustrates how interactions with an image dipole can modify the radiative properties of an emitter. We consider the specific example of an emitter positioned near the surface of a silver nanowire. The emitter radiates directly into the far field, but its local field also induces an image dipole in the nanowire. This image dipole acts as a secondary radiator that interferes with the emitter, thereby modifying both the intensity and the shape of the far-field emission.

In a nanostructure, the magnitude and orientation of the image dipole can be difficult to calculate, even for a simple wire geometry^32^. We can attain an intuitive understanding of the system in the limit in which the emitter is sufficiently close to the wire such that the surface is approximately flat and the distance is small compared with the wavelength of light (the electrostatic limit). In this limit, the orientation of the emitter dipole moment determines the relative phase of the image dipole, as illustrated in the right inset of Fig. 1a. A dipole oriented perpendicular to the surface (red) is in phase with its image dipole, whereas a dipole parallel to the surface (blue) is out of phase. These two configurations lead to different interference conditions.

We use numerical finite-difference time-domain (FDTD) calculations to attain a more precise description of the system (see Methods). Fig. 1b shows the calculated diffraction spots arising from an isotropic emitter located 30 nm from a nanowire surface for the emitted polarization component oriented perpendicular (top) and parallel (bottom) to the wire axis (which we denote as the *y* axis). The intensity of the diffraction spot depends strongly on the polarization of the emitted light. Parallel-polarized emission is suppressed, whereas perpendicularly polarized emission is enhanced, in agreement with the electrostatic picture presented in Fig. 1a.

Figure 1b also shows the position of the emitter (filled circle) and the calculated centroid position of the diffraction spot (open circle). A variety of methods exist for calculating the centroid position^4^,^5^. Here we determine the centroid by fitting the calculated far-field image to a Gaussian point-spread function. The centroid position can be used to measure the emitter position with a precision as fine as 1–2 nm^8^. However, Fig. 1b shows that proximity to the nanowire displaces the diffraction spot, resulting in a shift of the centroid position relative to the actual position of the emitter. The direction of the displacement depends strongly on the polarization of the emitted light. The diffraction spot shifts towards the wire for perpendicularly polarized emission and away from the wire for parallel-polarized emission. A video of this simulation is provided in the Supplementary Video 1.

In Fig. 1c, we plot the calculated far-field intensities, *I*_⊥_ and *I*_||_, measured along the perpendicular and parallel polarization directions, respectively, as a function of the distance between the emitter and the wire surface. Near the wire, parallel-polarized emission is suppressed and perpendicularly polarized emission is enhanced. As the distance between the emitter and the nanowire increases, the emission intensities exhibit a damped oscillatory behaviour. The oscillations occur when the distance between the emitter and the image dipole approaches the wavelength of light. At these distances, the electrostatic picture is no longer valid and the image dipole picks up an additional phase due to the finite propagation time of light from the dipole to the wire^33^,^34^. This phase retardation changes the interference condition for the two polarizations. At a distance of 80 nm from the wire surface, the retardation phase becomes greater than *π*/2 and the parallel component switches from destructive to constructive interference becoming brighter than the perpendicular component.

The displacement of the diffraction spot relative to the emitter position also depends on the distance between the emitter and the wire surface. Due to the symmetry of the wire geometry, the diffraction spot shifts only along the direction orthogonal to the wire surface, which we label *x* (see axes in Fig. 1b). We denote *x*_0_ as the *x* coordinate of the emitter position and *x*_⊥_ and *x*_||_ as the *x* coordinates of the centroid position for perpendicular- and parallel-polarized emission, respectively. Fig. 1d shows the centroid displacements *x*_⊥_−*x*_0_ (red) and *x*_||_−*x*_0_ (blue) as a function of the emitter distance to the wire surface. Near the wire surface, *x*_⊥_ is displaced towards the wire and *x*_||_ is displaced away from the wire. As the distance between the emitter and the surface increases, the centroid positions exhibit a damped oscillatory behaviour similar to that of the intensities. Although we have focused on the specific case of a metal particle, image-dipole effects also occur in dielectric structures^18^,^34^. In Supplementary Fig. 1 we plot the intensity and centroid displacement for dielectric nanowires with varying indices of refraction. High-index dielectrics (*n*>2.5) exhibit the most pronounced effects, which are similar in magnitude to those of a silver nanowire with the same dimensions.

### Explanation of the displacement of the diffraction spot

**Figure 2:**
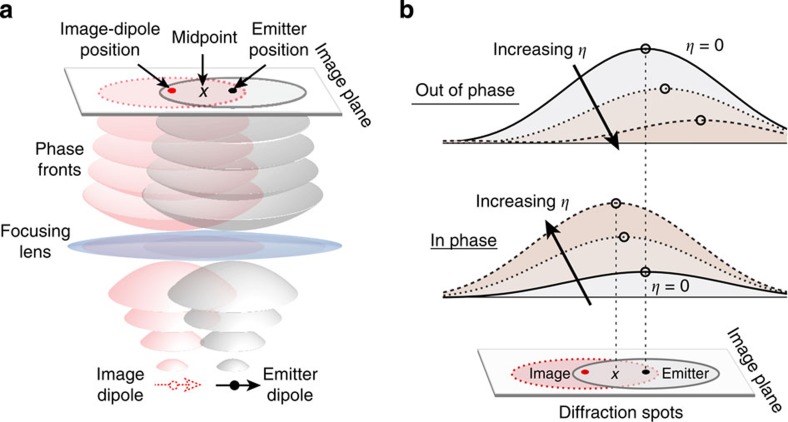
Young’s interference effect. (**a**) Diagram of the interference between the emitter dipole and the image dipole, which behave similarly to the slits in a Young’s double-slit experiment. Wave interference of the overlapping diffraction spots occurs at the image plane. (**b**) Calculated profiles of the diffraction pattern at the image plane along the *x* coordinate as a function of *η* (the ratio of the magnitude of the image dipole moment to the magnitude of the emitter dipole moment) when the image dipole is out of phase or in phase with the emitter dipole.

The image-dipole picture provides a simple explanation for the displacement of the diffraction spot, as illustrated in Fig. 2a. The emitter dipole and its image act as point-like sources that play roles analogous to the slits in a Young’s double-slit experiment. Because the distance between the dipoles is small compared with the diffraction spot size, their focused wavefronts overlap spatially and interfere at the image plane. This interference will distort the resulting diffraction spot, which can appear displaced from its original position. The amount and the direction of this displacement depend on the relative amplitude and phase of the light coming from the two sources. To demonstrate this dependence, we calculate the diffraction spot arising from two dipoles co-oriented and separated by a distance of 100 nm along the *x* direction. Fig. 2b shows the calculated diffraction spot along the *x* coordinate when the dipoles are in phase and out of phase. We plot diffraction patterns for various values of the ratio *η*=|*p*_*i*_|/|*p*_*e*_|, where |*p*_*e*_| is the magnitude of the emitter dipole moment and |*p*_*i*_| is the magnitude of the image dipole moment.

For *η*=0 (solid black lines), the in-phase and out-of-phase diffraction patterns coincide with the position of the emitter dipole. As *η* increases, the diffraction spots of the in-phase dipoles exhibit constructive interference that enhances the intensity in the region between the two dipoles where their diffraction spots overlap. Therefore, the centroid position (open circles) shifts towards the image-dipole position and the emission intensity increases. In contrast, when the image dipole is out of phase, the two emissions interfere destructively, so the centroid position shifts away from the image dipole and the emission intensity becomes weaker. Although we performed the above analysis for dipoles oriented along the *x* direction, the same behaviour occurs for dipoles oriented in the *y* and *z* directions as well (Supplementary Fig. 2).

When the emitter is close to the nanowire, interference with the image dipole shifts *x*_⊥_ towards the nanowire and *x*_||_ away from it. As the distance between the emitter and the nanowire increases beyond the electrostatic limit, the image dipole begins to oscillate with a phase retardation given by the finite propagation time of light. The accumulation of phase retardation with separation distance causes *x*_⊥_to smoothly transition from being pulled towards the wire to being pushed out from the wire (and vice versa for *x*_||_), resulting in the sinusoidal oscillations observed in Fig. 1d. We note that increasing the distance between the emitter and image dipole can also affect the interference condition and is equivalent to increasing the distance between the two slits in a Young’s double-slit interferometer. However, as we show in Supplementary Fig. 3, increasing the distance alone without changing the relative phase between the two dipoles will not change the direction (that is, the sign) of the displacements. It will only change the magnitude and thus cannot create the oscillatory behaviour shown in Fig. 1d.

### Polarization-resolved tracking

**Figure 3:**
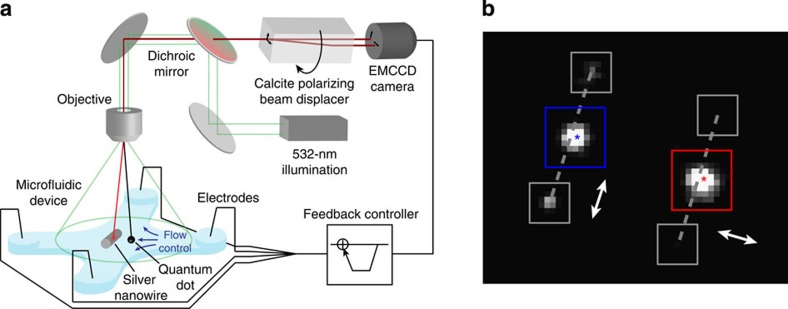
Polarization-resolved tracking. (**a**) Schematic of the experimental setup. Flow control in a microfluidic device is used to manipulate a quantum dot in the vicinity of an immobilized silver nanowire. The quantum dot is excited with a 532-nm laser. A calcite polarizing beam displacer separates the quantum-dot emission into polarizations that are perpendicular and parallel to the wire axis. These polarizations are imaged simultaneously on an electron-multiplying charge-coupled device (EMCCD) camera, and the quantum-dot centroid position is used to perform feedback flow control. (**b**) Image of a quantum dot coupling to a silver nanowire as seen along perpendicular (red) and parallel (blue) polarization directions relative to the wire axis (grey dashed lines). The polarization direction is also indicated by the double arrows. Boxes denote the pixel windows used to track the intensity and measure the centroid positions of the dot and nanowire emission.

We use polarization-resolved tracking to measure the interference effects described in the previous sections. We probe a silver nanowire with a single CdSe-ZnS quantum dot that acts as a point-like dipole emitter. We measure the emission of the quantum dot along two orthogonal polarizations simultaneously. Fig. 3a shows the experimental setup. We position the quantum dot near the silver nanowire with nanoscale precision using microfluidic flow control^9^,^35^,^36^ (see Methods for the details of experimental setup and technique). Although the emission of CdSe-ZnS quantum dots can exhibit some polarization dependence^37^, in our case, rapid orientational Brownian motion averages away any preferred polarization direction. A suspended quantum dot therefore behaves like an isotropic emitter (see Supplementary Fig. 4).

We excite the quantum dot with a 532-nm laser and collect its emission at 620 nm. We use a birefringent calcite crystal to generate two displaced images at orthogonal polarizations, and project both images onto the same camera. We adjust the calcite prism orientation so that one image is polarized perpendicular to the wire axis and the other is polarized parallel. Fig. 3b shows a double image for a single quantum dot positioned close to a silver nanowire. The quantum dot couples to the surface plasmon-polariton mode of the wire that waveguides a fraction of the emission to the wire ends^38^. The emission from the wire ends is polarized parallel to the wire axis, as expected for a single-mode wire^39^. We do not observe any emission along the length of the wire because guided surface plasmon polaritons do not radiate to the far field^40^.

### Mapping polarization anisotropy

To quantify the effect of the nanowire on the emitter polarization, we measure the polarization anisotropy, defined as *A*=(*I*_⊥_−*I*_//_)/(*I*_⊥_+*I*_//_). Emission polarized along the perpendicular and parallel directions give *A*=1 and *A*=−1, respectively, whereas unpolarized emission gives *A*=0. The polarization anisotropy is insensitive to quantum-dot blinking^41^ and local field enhancement of the pump^26^.

**Figure 4:**
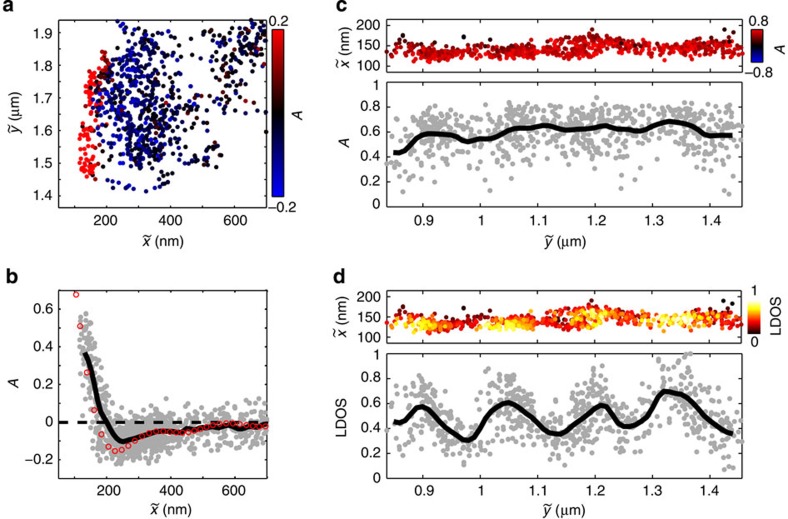
Polarization anisotropy and LDOS. (**a**) Scatter plot of *A* as a function of 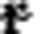. The axis of the wire is at 
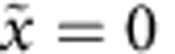
. (**b**) *A* as a function only of 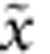 (grey circles). The black line is a running average and the red circles are values obtained from FDTD calculation. (**c**) Scatter plot of polarization anisotropy *A* as a function of 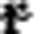 (coloured data points) and as a function only of 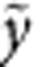 (grey data points). The black line is a running average of the data. (**d**) Scatter plot of the LDOS as a function of 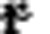 (coloured data points) and as a function only of 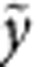 (grey data points) with black line running average.

Figure 4a shows the measured value of *A* as a function of the position 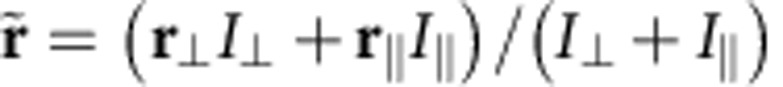, where **r**_⊥_ and **r**_||_ are the centroid positions of the quantum-dot diffraction spots measured along the perpendicular and parallel polarizations, respectively. The position 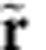 is the centroid of the diffraction spot in the absence of polarization optics (see Supplementary Note 1). We define 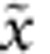 and 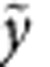 as the Cartesian components of 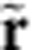, where 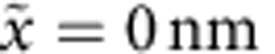 delineates the wire axis. When the quantum dot is far from the wire, the anisotropy approaches zero, corresponding to unpolarized emission. Near the nanowire, the quantum-dot emission is polarized perpendicular to the wire axis resulting in a positive anisotropy, in agreement with the interference illustrated in Fig. 1. Figure 4b is a plot of *A* as a function of 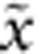 only. The black line is a Gaussian average of the data, where we set the standard deviation of the Gaussian filter to 12 nm, which corresponds to the finite spatial precision of the tracking algorithm^9^. The anisotropy decreases rapidly as the distance between the dot and the wire increases, and reverses sign at 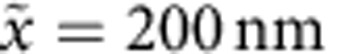. It reaches a minimum value of −0.1 at 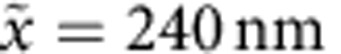, and decays to zero at long distances. The measured values agree well with the anisotropy obtained from FDTD calculation (red circles), which also incorporates the experimental spatial uncertainty (see Methods).

In addition to image-dipole interference, the measured polarization anisotropy might also arise from non-radiative coupling to guided surface plasmon-polariton modes^42^. We can rule out this possibility by mapping the polarization anisotropy and the rate of spontaneous emission into the guided surface plasmon modes of the nanowire simultaneously. This rate is often expressed in terms of the LDOS. We have previously shown that we can map out the LDOS by measuring the intensity emitted by the nanowire end and normalizing it to the total intensity emitted by the dot (summed over both polarizations)^9^.

Figure 4c,d show the measured values of *A* and the LDOS, respectively, from a second data set in which we probe a single quantum dot along the length of a wire. We use a continuous fluid flow along the direction orthogonal to the wire axis to position the dot as close as possible to the nanowire so that we can sample the high LDOS region. The LDOS exhibits a sinusoidal variation along the length of the wire (Fig. 4d) because backward propagating surface plasmon polaritons can reflect from the wire end and interfere with the forward propagating modes to create Fabry–Perot oscillations^9^,^40^. The nodes in the LDOS occur at positions where the reflected backward component interferes destructively with the forward component. A similar sinusoidal behaviour would appear in the anisotropy if it were caused by coupling to the guided surface plasmon polaritons. As shown in Fig. 4c, *A* does not exhibit any pronounced sinusoidal variation, indicating that the non-radiative coupling to surface plasmon polaritons does not affect the anisotropy significantly (see Supplementary Note 2 for further discussion).

### Mapping the displacement of the diffraction spot

**Figure 5:**
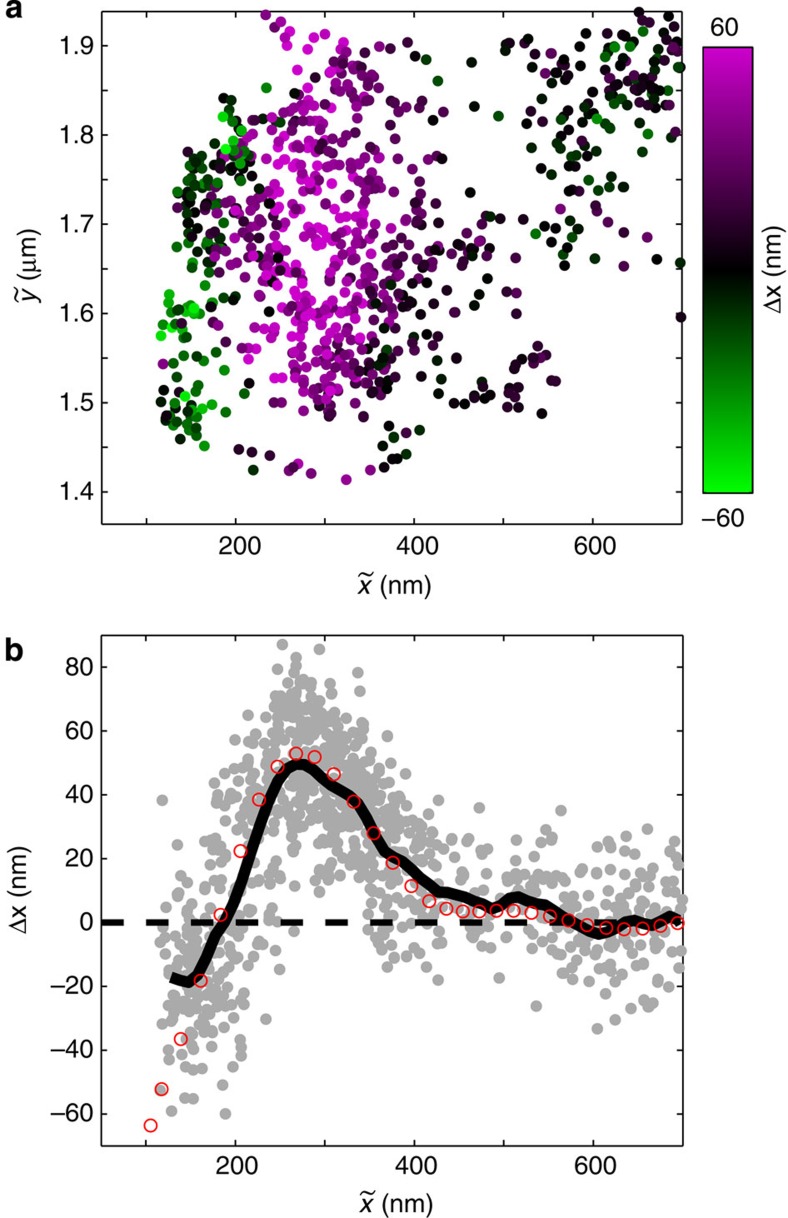
Displacement of the diffraction spot. (**a**) Scatter plot of the Δ*x* as a function of 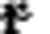. (**b**) Δ*x* as a function of 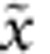, with a black line showing a running average of the data. The red circles are values obtained from FDTD calculations.

We use polarization-resolved tracking to measure the diffraction spot displacement caused by image-dipole effects. We measure the centroid position along both polarizations and define the relative centroid displacement as Δ*x*=*x*_⊥_−*x*_||_. Fig. 5a is a mapping of Δ*x* as a function of 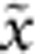 and 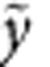 using the same data set used to produce Fig. 4a, and Fig. 5b plots Δ*x* as a function of 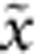 only. The black line is a Gaussian running average and the red circles show calculated values (both obtained in the same way as Fig. 4b). Near the wire, Δ*x* is negative indicating that the perpendicular component appears closer to the wire than the parallel component. As the distance between the quantum dot and the wire increases, the behaviour reverses before finally decaying to zero at long distances. We observe a maximum Δ*x* of 50 nm at 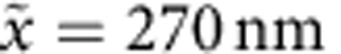. The experimental results agree well with the numerical calculation.

### Correcting image dipole-induced errors

The above results show that proximity to a nanostructure can substantially distort both the intensity and position of the far-field diffraction spot of an emitter. This effect should be taken into account in high-resolution single-emitter tracking applications. Polarization-resolved tracking provides additional information that can potentially be used to compensate for image-dipole effects to perform more accurate imaging. As a representative example, we use polarization-resolved tracking to map the LDOS of a silver nanowire along its radial direction.

**Figure 6:**
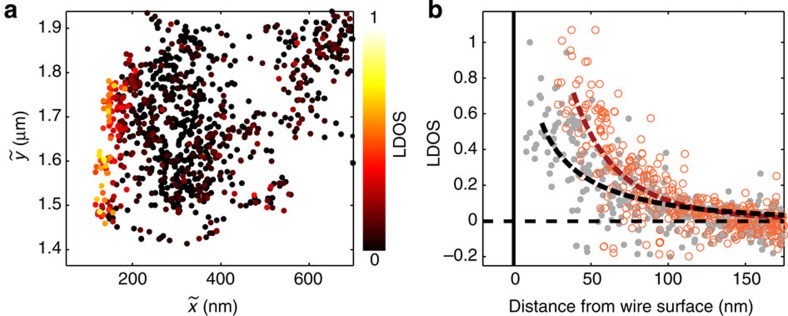
Correction of image-dipole errors in the measured LDOS. (**a**) Scatter plot of the LDOS. (**b**) Plot of LDOS as measured (grey circles with dashed black Bessel function fit) and corrected for image-dipole effects (red circles with dashed red Bessel function fit) as a function of the distance from the wire surface.

Figure 6a is a plot of the measured values of the LDOS as a function of 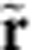 using the same data set that was used to produce Figs 4a and 5a. Near the wire surface, image-dipole effects distort both the measured intensity and centroid position. We can account for these effects by calculating the one-to-one relationship between the measured position 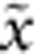 and the actual position *x*_0_ of the quantum dot. By inverting this relation, we can correct for the displacement of the diffraction spot and the change in emission intensity. To perform the inversion, we need to know the position of the wire surface. The interferometric oscillations of the centroid displacement Δ*x*=*x*_⊥_–*x*_||_ provide this information because they depend on the phase between the emitter and its image dipole. This phase is directly proportional to the distance of the emitter from the wire surface. We can therefore obtain the location of the wire surface by using the results of Fig. 5b to identify the surface position that leads to best agreement between the measured interference fringe (black curve) and the calculated fringe (red circles). The fitted surface position is 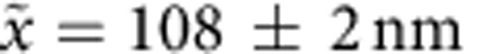 (uncertainty given as the 95% confidence bound). This position provides an absolute reference to calculate *x*_0_ from 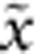 and correct the distortion to the centroid position. Once we know 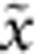, we can invert the distortion in intensity at that position. Supplementary Note 3 and Supplementary Fig. 5 provide a detailed description of the correction procedure. We note that the correction method we use here relies on knowledge of the geometry of the wire, which simplifies the inversion between the actual and measured positions. The approach can be extended to more general device geometries by casting it as an inverse problem and optimizing the device structure that provides best agreement with the measured displacement.

Figure 6b plots the uncorrected (grey) and corrected (red) values of the LDOS as a function of the absolute distance from the wire surface. We fit the decay of the LDOS profiles to a function of the form |*βK*_0_(*x*/*α*)|^2^, where *K*_0_(*x*) is the zeroth-order modified Bessel function^43^ and *α* and *β* are fitting parameters. The dashed lines in Fig. 6b are the resulting fits. The decay length parameter *α* for the uncorrected LDOS is 251±30 nm (all uncertainties given here are 95% confidence bounds), compared with 113±9 nm for the corrected LDOS. Thus, there is a significant difference between the corrected and uncorrected values. We compare these measurements with the numerically predicted value based on FDTD calculations (Supplementary Fig. 6), which gives *α=*127±12 nm. The decay parameter determined by the corrected LDOS provides much better agreement with the theoretically predicted value. The displacement of the centroid position and the modification of the radiated intensity make the uncorrected LDOS appear to decay more gradually than it actually does, resulting in a decay constant that is off by nearly a factor of 2.

## Discussion

In summary, we have demonstrated that the interference between an emitter and its image dipole can induce substantial polarization anisotropy and significant displacement of the emitter’s diffraction spot. By using polarization-resolved tracking, we were able to make direct measurements of the effect of an image dipole on the radiation of an emitter near a silver nanowire. We observed significant spatial displacements of the diffraction spot even at distances exceeding 200 nm from the nanowire. We explained the large displacements in the centroid position as a Young’s interference effect between the quantum dot and its image dipole. By correcting for this effect, we achieved an improved measurement of the LDOS that is in good agreement with theoretical calculations.

Our results have important implications for imaging and sensing applications that use single-emitter tracking^8^,^9^,^10^,^11^,^12^,^13^,^14^. We have shown that spatial displacements of the diffraction spot can be as large as 50 nm, which could create substantial tracking errors. Although our results focused on the specific example of a silver nanowire, image-dipole effects can also arise in dielectric structures^18^,^34^. Rather than solely being a source of error, however, the diffraction spot displacement also carries useful information. This displacement can be used to develop better insight to image dipole effects and can potentially improve the spatial accuracy for measures of important quantities such as the LDOS. These results open up new possibilities for performing high-accuracy imaging and sensing near surfaces. Ultimately, our results could provide a pathway for the accurate probing and study of nanoscale systems using single-emitter tracking, as well as for controlling interactions between nanoscopic systems.

## Methods

### FDTD calculations

FDTD calculations were performed with the Lumerical FDTD software package ( http://www.lumerical.com). For all calculations, we assumed a background refractive index of 1.40 surrounding a silver nanowire (4 μm long and 100 nm diameter). We simulated the radiation patterns of dipoles oriented perpendicular, parallel and vertically out of plane with respect to the axis of the nanowire and at different distances from the wire’s surface. The far-field response was obtained by first measuring the electric fields in the near field and then performing a far-field projection. We retained only collection angles that fit within an NA of 1.45 (consistent with the NA used in the experiments), and propagated the fields to the image plane using a Fourier transform. The far-field intensities from each of the three dipole orientations measured along the perpendicular or parallel polarizations were added together to model radiation from an isotropic emitter. The intensity and centroid position of the far-field diffraction spot were obtained by summing the intensity of the diffraction spot and fitting it to a two-dimensional Gaussian point-spread function, respectively. In Figs 4b and 5b, we spatially convolve the simulated curves with a normalized Gaussian function with a 12 nm s.d. so that it can be directly compared with the experimental results that are averaged with the exact same Gaussian filter. The location of the wire surface was used as a fitting parameter to optimize the agreement with the experimentally obtained curve in Fig. 5b. This fit accounts for the fact that we do not know the precise location of the wire surface in the experimentally measured data. The fitted surface position obtained from Fig. 5b is used consistently throughout the manuscript where the simulation is compared with experimental data.

### Procedure for microfluidic control of quantum dots

The microfluidic device consisted of a moulded PDMS cross-channel placed on top of a glass coverslip. Silver nanowires were synthesized in solution using a technique previously reported by Sun *et al.*^44^, and were deposited on the top PDMS channel surface prior to filling the channel with the quantum-dot fluid. The synthesized silver nanowires were several microns in length and 100–120 nm in diameter. We dispersed colloidal quantum dots (Ocean NanoTech, carboxylic acid) in a water-based fluid, which was introduced into the channel prior to the experiment. Full details of the fluid chemistry were reported in the previous work^9^. This fluid chemistry creates a thin water sheath that adsorbs along the channel walls when filled into the microfluidic device. The quantum dots are confined within this sheath and localized to within 100 nm of the channel surface.

Quantum dots were positioned using electrodes to actuate electroosmotic motion of the fluid across the channels^35^. We used a 1.45-NA, oil-immersion objective to illuminate and image single quantum dots, which were excited by a linearly polarized laser at an intensity of 250 W cm^−2^ and a wavelength of 532 nm. The emission polarization of the quantum dots was found to be independent of the pump polarization. The laser was focused to a spot of ~10 μm diameter, so that only quantum dots near the target nanowire were illuminated. We used a bandpass filter centreed at the quantum dot peak emission wavelength (620 nm) to image their emission on an electron multiplying charge-coupled device camera.

By optically tracking the position of a suspended quantum dot in real time, we performed feedback control of the flow to achieve two-dimensional positioning of the emitter within the central control region of the microfluidic device. Details of the microfluidic positioning system have been reported previously^35^. We used the centroid position of the quantum dot imaged in the parallel polarization for feedback control. For each camera frame we imaged the quantum dot and the nanowire at the polarizations perpendicular and parallel to the wire axis. We monitored six diffraction spots in each frame: two images of the quantum dot and four images of the nanowire ends. The intensity of each spot was determined by summing the pixel intensities within the square tracking region (shown in Fig. 3b) and subtracting a background level that was determined by summing the intensity from pixels just outside the tracking regions. Centroid positions were obtained with subwavelength localization by fitting each diffraction spot image to a two-dimensional Gaussian point-spread function. Every centroid position of the quantum dot was calculated relative to the bottom wire end and the wire’s axis, with the axis of the wire defined by the line connecting the two wire ends.

## Author contributions

C.R., E.W. and J.T.F. conceived the idea for the paper; Z.C. wrote software for the experiment and C.R. performed the experiments and analysed the data; S.N. synthesized the nanowires; and C.R., E.W., B.S. and J.T.F. contributed to writing the paper.

## Additional information

**How to cite this article:** Ropp, C. *et al.* Nanoscale probing of image-dipole interactions in a metallic nanostructure. *Nat. Commun.* 6:6558 doi: 10.1038/ncomms7558 (2015).

## Supplementary Material

Supplementary Figures, Supplementary Notes and Supplementary References Supplementary Figures 1-7, Supplementary Notes 1-3 and Supplementary References

Supplementary Movie 1 Video of the simulated far-field image of an isotropic emitter as it is positioned near a silver nanowire with 100 nm diameter (white outline). The top panel is the perpendicular polarized image and the bottom is the parallel polarized image. In each frame, the black dot corresponds to the emitter's actual position and the black open circle corresponds to the centroid position obtained by fitting the far-field image to a two-dimensional Gaussian point spread function. The vertical black line delineates the x position of the emitter.
