# Supplementary material for: Nanoscale probing of image-dipole interactions in a metallic nanostructure
Source: Nat Commun. 2015 Mar 19;6:6558. doi: 10.1038/ncomms7558 (PMC4383001; doi:10.1038/ncomms7558)
Supplement: Supplementary Figures, Supplementary Notes and Supplementary References — Supplementary Figures 1-7, Supplementary Notes 1-3 and Supplementary References [file ncomms7558-s1.pdf]

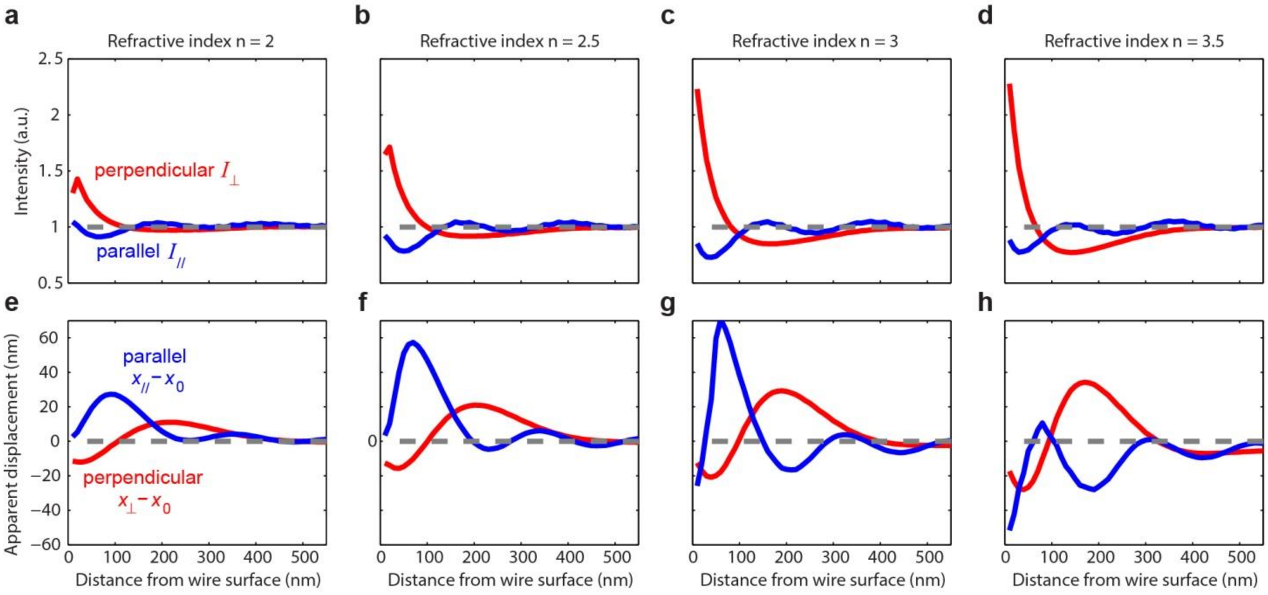

Supplementary Figure 1 | Image-dipole effects caused by a dielectric nanowire. (a-d) Calculated change in the far-field intensity of an isotropic emitter placed near a dielectric nanowire and measured along the polarization parallel and perpendicular to the nanowire axis. Intensity change is plotted as a function of distance from the wire surface and for different nanowire refractive indices. **(e-h)** Calculated displacement of the diffraction spot of an isotropic emitter measured along the parallel and perpendicular polarizations as a function of distance from the wire surface and for different nanowire refractive indices. For all calculations, we simulate a dielectric nanowire with a 100 nm diameter and a background refractive index of 1.4, the same parameters used to generate Fig. 1 in the manuscript for a silver nanowire. All panels show a clear oscillatory behavior similar to those attained for a silver nanowire. We note that within 80 nm from the wire surface, the diffraction spot measured along the parallel polarization is pulled into the wire. This behavior coincides with a strong distortion of the diffraction spot shape, which is likely caused by increased scattering from the air-dielectric interfaces. A complete understanding of these complex near-field effects in dielectric structures requires further investigation.

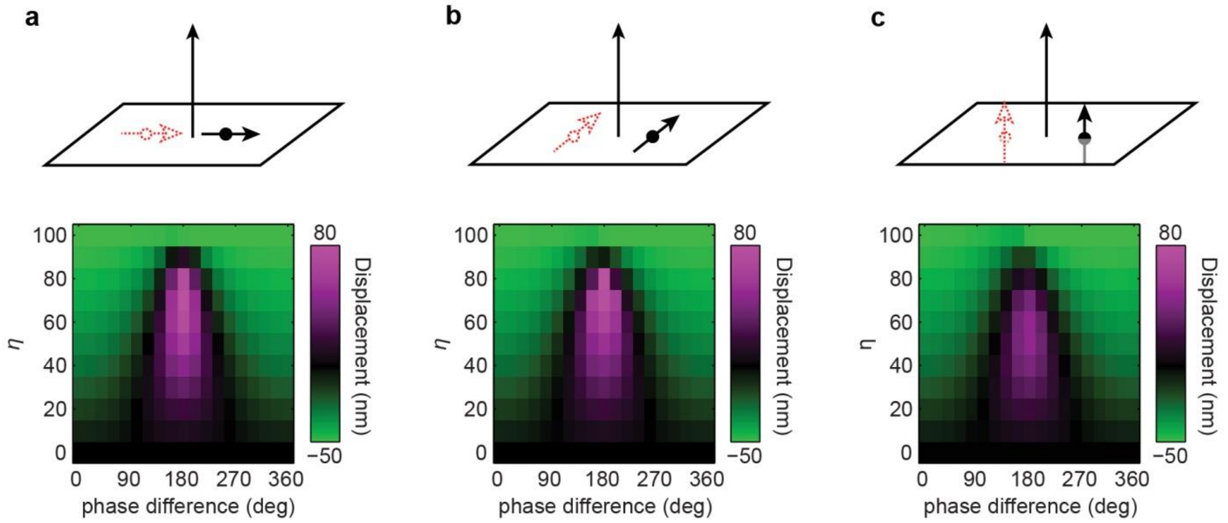

Supplementary Figure 2 | Diffraction spot displacement due to an image dipole. Displacement of the diffraction spot from the position of an emitter dipole (black dipole) plotted as a function of the relative phase and amplitude of the image dipole (red dipole) and shown for three different dipole orientations (geometry displayed above each panel). For each case the image dipole is co-oriented with the emitter dipole and located 100 nm away. The position of the diffraction spot is determined by measuring the centroid of the far-field image, taken along the vertical direction (as indicated by the black arrow). In the color plots, $\eta = |p_i|/|p_e|$, where p_e is the emitter dipole moment and p_i is the image dipole moment. For all orientations, the diffraction spot centroid position oscillates between displacement toward the image dipole (green) and displacement away from the image dipole (purple) as a function of the phase difference between the two dipoles. These oscillations only occur if the image dipole is weaker than the emitter dipole. If both dipoles have equal amplitude (η = 1) then the far-field diffraction spot will be symmetric and the centroid position will be located at the midpoint between the two dipoles (corresponding to a displacement of -50 nm).

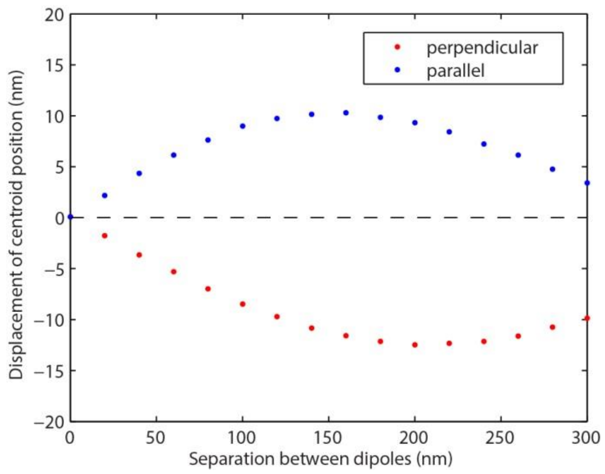

Supplementary Figure 3 | Displacement of the diffraction spot caused by dipole separation. Plot showing the displacement of the diffraction spot centroid for a system comprised of an emitter dipole and an image dipole whose amplitude is 10 times weaker than the emitter. This displacement is plotted as a function of the separation distance between the two dipoles. The red data points are calculated for a system where the two dipoles are oriented perpendicular to the image plane and held in phase with one another. The blue data points are calculated for a system where the two dipoles are oriented parallel to the image plane and held at 180° out of phase from one another. The in-phase system shows the diffraction spot is always pulled towards the image dipole, while the out-of-phase systems shows the diffraction spot is always pushed away from the image dipole. The direction of the displacement never reverses, in contrast to the clear oscillatory behavior observed in Fig. 1d of the manuscript.

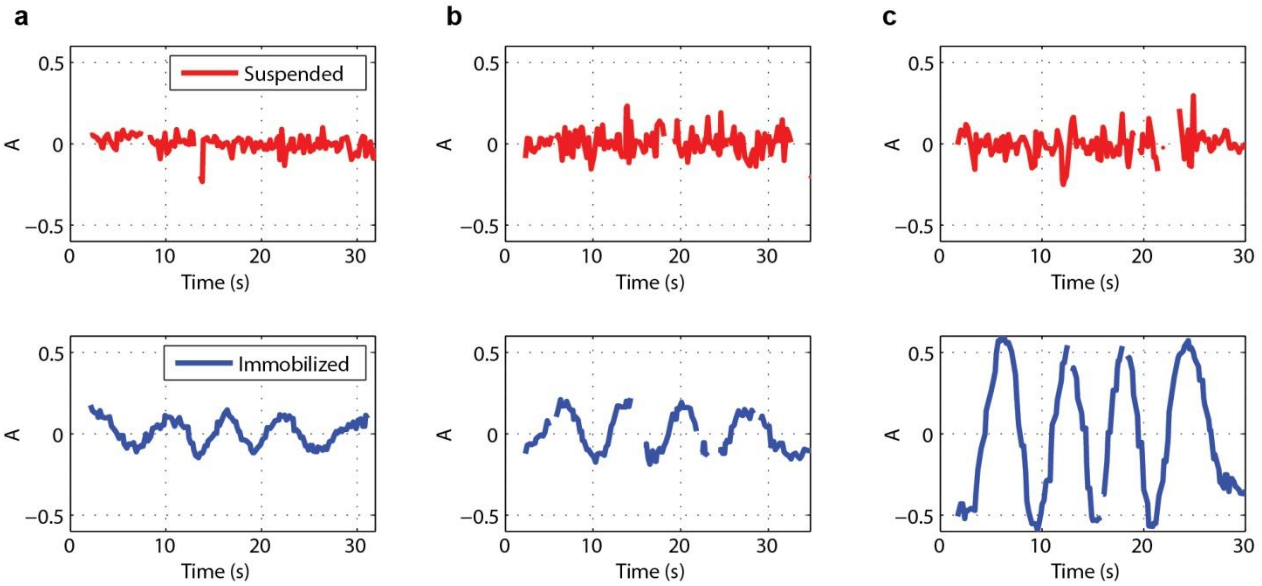

Supplementary Figure 4 | Comparing free-floating and immobilized quantum dot anisotropy. The anisotropy measured from three pairs of quantum dots, suspended (red, top curves) and immobilized (blue, bottom curves). The anisotropy is plotted as a function of the emission polarization as it is rotated in time using a half-wave plate, which is placed before the calcite prism in our experimental setup. The anisotropy is measured as $A = (I_{\mathrm{V}} - I_H)/(I_V + I_H)$, where I_V and I_H are the far-field intensities measured along the vertical and horizontal polarization directions, respectively. The immobilized quantum dots exhibit polarized emission, whereas the suspended quantum dots appear unpolarized, which we attribute to their fast rotational Brownian motion in the liquid that averages out polarization.

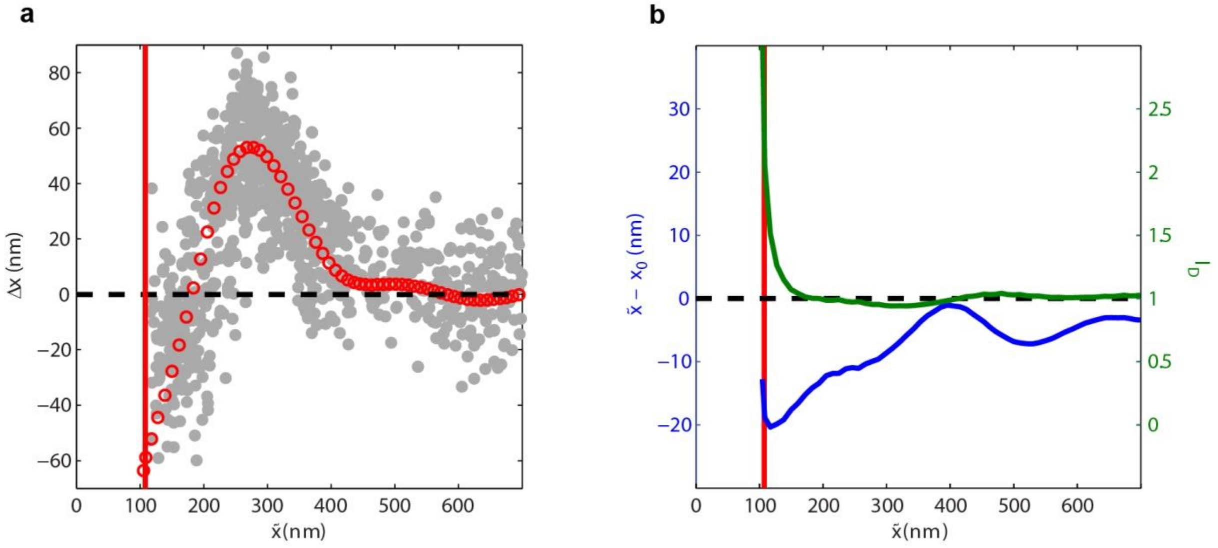

Supplementary Figure 5 | Compensation for image-dipole effects. (a) Plot of the polarization-dependent shift, Δx, as a function of $\tilde{x}$ for the experimental data (gray) and the simulated data (red). The location of the wire's surface is shown with the vertical red line. **(b)** FDTD calculation of the centroid position displacement (blue) and the intensity error, I_D, (green) as a function of $\tilde{x}$ and relative to the fitted location of the wire surface (red line)

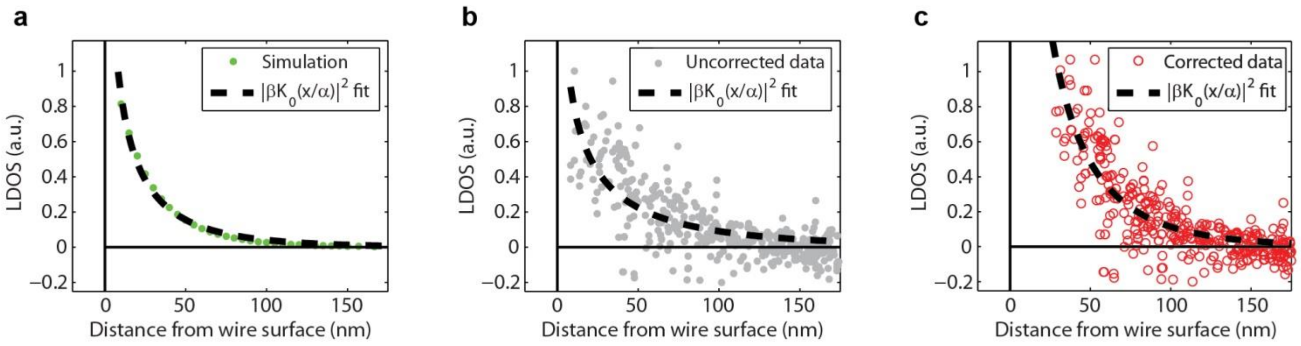

Supplementary Figure 6 | Comparison of LDOS fits to the ideal Bessel function decay. Plots of the FDTD simulated LDOS profile **(a)**, the uncorrected data **(b)**, and the corrected data **(c)** with corresponding fits to a Bessel function of the form $\left|\beta K_0(x/\alpha)\right|^2$. Here, $K_0(x)$ is the zeroth-order modified Bessel function, α is the decay length, and β is an overall scaling factor. Both α and β are treated as fitting parameters.

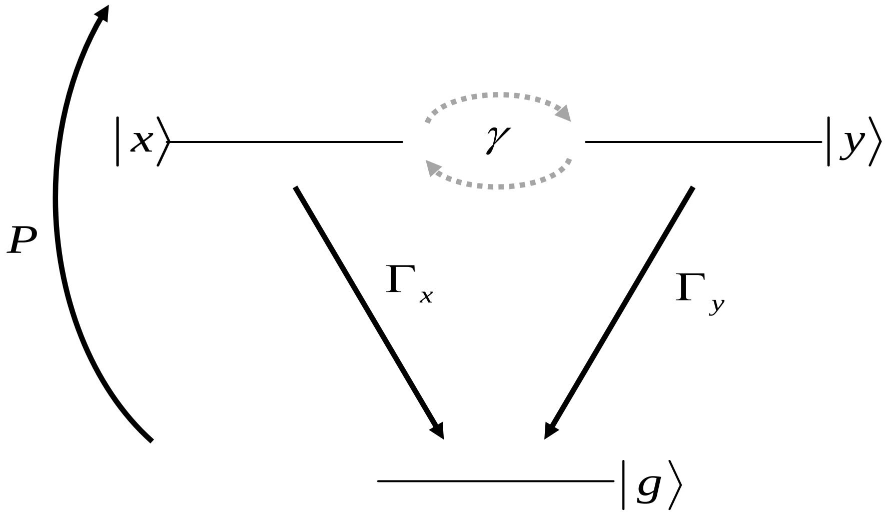

Supplementary Figure 7 | Energy level structure and decay rates for a CdSe quantum dot. State $|x\rangle$ and $|y\rangle$ are the bright exciton states whose dipole moment is oriented in the *x* and *y* direction. The bright exciton states decay with decay rates Γ_x and Γ_y to the quantum dot ground state $|g\rangle$. In addition, the bright excitons exhibit rapid randomization that incoherently couples the two exciton states with rate γ.

Supplementary Note 1

Calculation of the centroid position without polarization detection

In the main text we equate the centroid position of the quantum dot in the absence of polarization optics to:

$$\tilde{\mathbf{r}} = \left(\mathbf{r}_{\perp} I_{\perp} + \mathbf{r}_{//} I_{//}\right) / \left(I_{\perp} + I_{//}\right) , \qquad (1)$$

where $\mathbf{r}_{\perp}$ ($\mathbf{r}_{\parallel}$) is the centroid position obtained from fitting the diffraction spot image along the perpendicular (parallel) polarization to a two-dimensional Gaussian point spread function. In eq. 1, $\tilde{\mathbf{r}}$ is the intensity-weighted average of the centroids of the two polarized images.

The correspondence of the intensity-weighted average with the centroid in the absence of polarization optics can be seen from the definition of centroid position:

$$\mathbf{C} = \frac{\sum_i I_i \mathbf{r}_i}{\sum_i I_i} , \qquad (2)$$

where $\mathbf{r}_i$ and I_i are the position and intensity, respectively, of the ith data point. Combining the centroid positions from the two polarizations:

$$\begin{aligned}
\mathbf{C}_{com} &= \frac{\sum_i \left(I_i^{\perp}\mathbf{r}_i^{\perp} + I_i^{\parallel}\mathbf{r}_i^{\parallel}\right)}{\sum_i \left(I_i^{\perp} + I_i^{\parallel}\right)} \\
&= \frac{\sum_i I_i^{\perp}\mathbf{r}_i^{\perp}}{\sum_i \left(I_i^{\perp} + I_i^{\parallel}\right)} \left(\frac{\sum_i I_i^{\perp}}{\sum_i I_i^{\perp}}\right) + \frac{\sum_i I_i^{\parallel}\mathbf{r}_i^{\parallel}}{\sum_i \left(I_i^{\perp} + I_i^{\parallel}\right)} \left(\frac{\sum_i I_i^{\parallel}}{\sum_i I_i^{\parallel}}\right) \\
&= \frac{\mathbf{C}^{\perp}\sum_i I_i^{\perp} + \mathbf{C}^{\parallel}\sum_i I_i^{\parallel}}{\sum_i \left(I_i^{\perp} + I_i^{\parallel}\right)} \\
&= \frac{\mathbf{C}^{\perp} I^{\perp} + \mathbf{C}^{\parallel} I^{\parallel}}{I^{\perp} + I^{\parallel}} \qquad (3)
\end{aligned}$$

Supplementary Note 2

Anisotropy of far-field radiation in the presence of rapid spin relaxation

CdSe quantum dots exhibit a fast internal spin relaxation between the exciton states, which is on the order of 1 ps^{-1} [1,2]. This spin relaxation rate is much faster than the Purcell enhanced decay rate of the quantum dot into the nanowire, typically on the order of 8-12 ns for our system[3]. Spin relaxation will have a significant impact on the anisotropy of the far-field radiation, which we can analyze using the level structure shown in in Supp. Fig. 7 that represents a good quantum model for a CdSe quantum dot[4]. For simplicity, we consider a reduced level structure that includes only the lowest energy bright excitons and ignore dark states that are not radiatively active. The two bright exciton states $|x\rangle$ and $|y\rangle$ represent excitons whose dipole transitions are oriented along the *x* and *y* directions respectively. We note that in a spherically symmetric quantum dot we would typically express the level structure using the angular momentum basis $|+1\rangle$ and $|-1\rangle$. However, at room temperature the natural linewidth of the quantum dot (> 10 meV) is much broader than any hyperfine or anisotropic energy shifts, so these two states are energy degenerate to a very good approximation. Thus, we can choose to work with the linearly polarized dipole states $|x\rangle$ and $|y\rangle$ which are superpositions of the angular momentum states, but still represent good stationary states the system. We use the same definition of the *x* and *y* directions defined in the manuscript, where *x* denotes the direction normal to the wire and *y* the direction parallel to the wire.

We use a rate equation formalism to calculate the anisotropy as well as the emission from the wire end. We denote P as the pump rate of the quantum dot. The decay rates of the bright excitons to the ground state are denoted by $\Gamma_i = \Gamma_i^R + \Gamma_i^{NR}$ (i=x,y). Here, Γ_i^R is the decay rate due to radiative emission of a photon into the far-field, while Γ_i^{NR} is the decay rate into guided surface plasmon polariton modes of the nanowire. The decay rate Γ_i^R depends on image dipole interactions, but does not depend on the LDOS of the guided surface plasmon modes. In contrast, Γ_i^{NR} is directly proportional to this LDOS. In addition to these decay rates, the quantum dot also exhibits a rapid spin relaxation rate γ that incoherently flips one bright exciton to the other.

We first consider the case without the spin-flip process (γ=0). The rate equations for the two excited state populations are then,

$$\dot{N}_x = -\Gamma_x N_x + P$$
$$\dot{N}_y = -\Gamma_y N_y + P$$

where N_x and N_y represent the average occupation probabilities for state $|x\rangle$ and $|y\rangle$ respectively. In the above equation we have assumed that the pump rate is sufficiently weak so that the dot is not being saturated. The steady state solution gives $N_x = P/\Gamma_x$ and $N_y = P/\Gamma_y$, and radiated intensities into the

far field along the x and y polarizations are given by $I_x = \Gamma_x^R N_x = \eta_x P$ and $I_y = \Gamma_y^R N_y = \eta_y P$, where $\eta_x = \Gamma_x^R / \Gamma_x$ and $\eta_y = \Gamma_y^R / \Gamma_y$ are the fraction of quantum dot excitations emitted into the x and y polarization respectively. From these two intensities we define the emission anisotropy as $A = (I_x - I_y)/(I_x + I_y) = \left(\eta_x - \eta_y\right)/\left(\eta_x + \eta_y\right)$. Since η_x and η_y depend on both image dipole effects (through Γ_i^R) and the LDOS (through Γ_i^{NR}) we expect a complex interplay between these two effects.

We next analyze the case where the two excited states have a fast spin relaxation term, and the rate equations become:

$$\dot{N}_x = -\Gamma_x N_x - \gamma(N_x - N_y) + P$$
$$\dot{N}_y = -\Gamma_y N_y + \gamma(N_x - N_y) + P$$

Solving for the steady state solution gives $N_x = P(\Gamma_y + 2\gamma)/(\Gamma_x\Gamma_y + \gamma(\Gamma_x + \Gamma_y))$ and $N_y = P(\Gamma_x + 2\gamma)/(\Gamma_x\Gamma_y + \gamma(\Gamma_x + \Gamma_y))$, which in the limit that $\gamma >> \Gamma_1, \Gamma_2$ results in $N_1 = N_2 = P/\overline{\Gamma}$, where $\overline{\Gamma} = \left(\Gamma_x + \Gamma_y\right)/2$. As a result, the emission anisotropy becomes $A = (\Gamma_1^R - \Gamma_2^R)/(\Gamma_1^R + \Gamma_2^R)$, which depends only on the radiative decay into the far-field, and is independent of non-radiative decay to the propagating SPPs in the nanowire. Since Γ_i^R does not depend on the LDOS but is modified by image dipole effects, the anisotropy now directly maps image dipole effects only. The above model is now fully consistent with Fig. 4a as well as Fig. 4c of the manuscript, which show that the anisotropy increases near the nanowire and there is no dependence on the anisotropy as we scan the quantum dot along the wire. The emission intensity from the wire end is given by $I_W = \Gamma_x^{NR} N_x + \Gamma_y^{NR} N_y$. Since Γ_i^{NR} is proportional to the LDOS, we expect to see a strong modulation of the intensity of the wire end as we scan the QD across the wire. Thus, the model also fully explains the data in Fig. 4d of the manuscript.

Supplementary Note 3

Procedure for compensating image-dipole effects

We compensate for image-dipole effects in our experimental data by using the information obtained from polarization-resolved tracking. The measured polarization-dependent shift vector $\Delta\mathbf{r} = \mathbf{r}_{\perp} + \mathbf{r}_{\parallel}$ and the centroid position $\tilde{\mathbf{r}}$ together provide a reference frame for calculating the absolute distance to the wire surface. We determine this reference frame by aligning the experimentally measured interference fringe for Δx with the fringe expected from FDTD calculations (shown together in Supp. Fig. 5a). The two data sets are aligned by using the location of the wire surface (red line in Supp. Fig. 5a) as a fitting parameter to minimize least squares differences between the simulated and experimental fringes.

Once the fringes are aligned, we obtain a one-to-one correspondence between the centroid position $\tilde{x}$ and the actual position x_0. This correspondence is shown as a blue curve in Supp. Fig. 5b, which plots $\tilde{x} - x_0$ as a function of the measured position $\tilde{x}$. This function has been translated using the location of the wire surface (red curve in Supp. Fig. 5a) and aligned with the experimental reference frame. To correct for centroid displacement in our experimental data, we translate each data point by the amount indicated on the blue curve given its centroid position. Similarly, we compensate for intensity error using the green curve plotted in Supp. Fig. 5b. This curve shows the normalized intensity error $I_D = [I_{\perp}(x_0) + I_{//}(x_0)]/[I_{\perp}(\infty) + I_{//}(\infty)]$, where $I_{\perp}(\infty)$ ($I_{//}(\infty)$) is the measured intensity in the perpendicular (parallel) polarization direction when the emitter is far away from the wire. Using its centroid position, we scale the measured intensity for each data point by a factor equal to 1 / I_D to perform the correction.

Supplementary References

1. Scholes, G. D. *et al.* Nanocrystal shape and the mechanism of exciton spin relaxation. *Nano Lett.* **6,** 1765–1771 (2006).
2. Kim, J. *et al.* Mechanism and origin of exciton spin relaxation in CdSe nanorods. *J. Phys. Chem. B* **110,** 25371–25382 (2006).
3. Ropp, C. *et al.* Nanoscale imaging and spontaneous emission control with a single nano-positioned quantum dot. *Nat. Commun.* **4,** 1447 (2013).
4. Norris, D. J., Efros, A. L., Rosen, M. & Bawendi, M. G. Size dependence of exciton fine structure in CdSe quantum dots. *Phys. Rev. B* **53,** 16347–16354 (1996).
